# Splicing control by PHF5A is crucial for melanoma cell survival

**DOI:** 10.1111/cpr.13741

**Published:** 2024-08-30

**Authors:** Tina Meißgeier, Melanie Kappelmann‐Fenzl, Sebastian Staebler, Ata Jadid Ahari, Christian Mertes, Julien Gagneur, Lisa Linck‐Paulus, Anja Katrin Bosserhoff

**Affiliations:** ^1^ Institute of Biochemistry Friedrich‐Alexander‐University Erlangen‐Nürnberg (FAU) Erlangen Germany; ^2^ Faculty of Computer Science Deggendorf Institute of Technology Deggendorf Germany; ^3^ School of Computation, Information and Technology Technical University of Munich Garching Germany

## Abstract

Abnormalities in alternative splicing are a hallmark of cancer formation. In this study, we investigated the role of the splicing factor PHD finger protein 5A (PHF5A) in melanoma. Malignant melanoma is the deadliest form of skin cancer, and patients with a high PHF5A expression show poor overall survival. Our data revealed that an siRNA‐mediated downregulation of PHF5A in different melanoma cell lines leads to massive splicing defects of different tumour‐relevant genes. The loss of PHF5A results in an increased rate of apoptosis by triggering Fas‐ and unfolded protein response (UPR)‐mediated apoptosis pathways in melanoma cells. These findings are tumour‐specific because we did not observe this regulation in fibroblasts. Our study identifies a crucial role of PHF5A as driver for melanoma malignancy and the described underlying splicing network provides an interesting basis for the development of new therapeutic targets for this aggressive form of skin cancer.

## Graphical abstract

This work investigates the role of the splicing factor PHD Finger Protein 5A (PHF5A) in melanoma progression. PHF5A is upregulated in melanoma cells, leading to an increased splicing rate, which ensures the tumour typical high proliferation rate and apoptosis resistance. An inhibition of PHF5A results in splicing defects, inhibited proliferation and increased apoptosis by UPR activation.

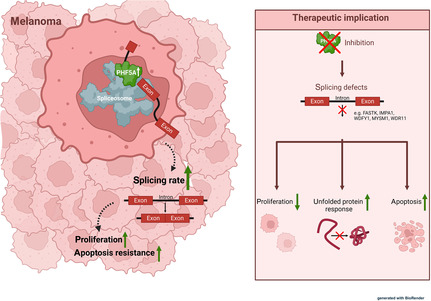

## 1 INTRODUCTION

Because of its high rate of metastasis and continuously increasing incidence, malignant melanoma is one of the most dangerous and aggressive cancer types.^1^ It arises from melanocytes, the pigment‐producing cells, which are mainly found in the skin, but can also occur in other tissues.^2^ As one of the most frequent risk factors, prolonged UV‐radiation causes DNA damage and mutations in skin cells, fostering malignant tumour development.^1^ Despite remarkable progress in targeted melanoma therapy over the past decades, resistance to inhibitors remains a significant challenge.^3^

One reason for resistance against therapeutic drugs is alternative splicing of tumour‐relevant genes. For example, the gene *BRAF*, which carries an activating mutation in about 50% of melanoma,^4^ occurs as a splice variant lacking exon 4–8, resulting in a loss of the RAS‐binding domain and therefore resistance against respective inhibitors.^5^ RNA‐splicing is a crucial step in the regulation of eukaryotic gene expression. It is an important player in reaching high genetic diversity; however, its dysregulation is associated with the development and progression of multiple types of cancer.^6^, ^7^, ^8^ Because tumours exhibit about 30% more alternative splicing events than non‐tumour cells, alternative splicing is a significant molecular characteristic of human cancer. Due to this enhanced rate of splicing, the molecular mediators of this process appear to be interesting candidates for therapeutic intervention.^9^, ^10^ It is known that several splicing factors are upregulated in cancer cells, promoting tumour. For example, as part of the spliceosome, BUD31 was shown to be upregulated in ovarian cancer, which is associated with a bad prognosis.^11^ The inhibition of BUD31 leads to splicing defects including substantial exon skipping and production of shortened isoforms, which can lead to the promotion of ovarian cancer.

RNA splicing is a dynamic process, canonically catalysed by the spliceosome, a 13‐megadalton protein complex, which contains five small nuclear ribonucleoprotein particle subunits (U1, U2, U4, U5 and U6 snRNP) and a number of other proteins.^12^

PHD finger protein 5A (PHF5A) is part of the U2 snRNP and is involved in the recognition of branch point regions during the splicing process.^13^ PHF5A is a highly conserved protein consisting of 110 amino acids, which belongs to the PHD‐finger superfamily.^14^ Besides splicing, PHF5A is further involved in chromatin remodelling,^14^ DNA repair,^15^ and other biological processes, such as cell cycle regulation,^16^ or regulation of pluripotency and differentiation of embryonic stem cells.^17^ Recent studies have linked an upregulation of PHF5A with the development of different cancer types, such as lung adenocarcinoma, breast cancer, and glioblastoma.^18^, ^19^, ^20^ However, the role of PHF5A regarding development and progression of malignant melanoma remains unknown. This study identifies PHF5A as a putative oncogene in malignant melanoma.

## 2 MATERIALS AND METHODS

### 2.1 Cell culture

Melanoma cell lines MelHo (RRID: CVCL_1402), 501Mel (RRID: CVCL_4633) and MelJuso (RRID: CVCL_1403) were cultivated in RPMI 1640 medium (Sigma‐Aldrich Chemie GmbH, Steinheim, Germany), which was supplemented with 2% sodium bicarbonate. For MV3 (RRID: CVCL_W280), DMEM high‐glucose medium (Sigma‐Aldrich Chemie GmbH, Steinheim, Germany) was used. The cell lines MelEi (RRID: CVCL_3978), MelIm (RRID: CVCL_3980), MelJu (RRID: CVCL_3979), MelWei (RRID: CVCL_3981) and SKMel28 (RRID: CVCL_0526) were cultivated in DMEM low‐glucose medium (Sigma‐Aldrich Chemie GmbH, Steinheim, Germany). Human fibroblasts (RRID: CVCL_3653) were cultivated in DMEM high‐glucose medium and obtained from Dr. Ingo Thievessen (Biophysics, Center for Medicine, Physics and Technology, FAU Erlangen‐Nürnberg, Erlangen, Germany). To each cell culture medium, 10% foetal bovine serum (FBS) (Sigma‐Aldrich Chemie GmbH, Steinheim, Germany) and 1% penicillin/streptomycin (Sigma‐Aldrich Chemie GmbH, Steinheim, Germany) were added. The cells were cultivated at 37°C and 8% CO_2_. The cell lines WM1158 (RRID: CVCL_6785), WM1366 (RRID: CVCL_6789), WM3211 (RRID: CVCL_6797) and Sbcl2 (RRID: CVCL_D732) were cultivated in tumour medium (TU: 80% MCDB152 (Sigma‐Aldrich Chemie GmbH, Steinheim, Germany), 20% Leibovitz L15 (Sigma‐Aldrich Chemie GmbH, Steinheim, Germany), 5 μg/mL Insulin, 1.68 mM CaCl2, 2% FBS, 1% penicillin/streptomycin). Normal human epidermal melanocytes (NHEM) were received from Lonza (Lonza Group AG, Basel, Switzerland) and cultivated in Lonza MGM‐4 BulletKit medium, containing 1% penicillin/streptomycin. Cells cultivated in tumour medium or MGM‐4 BulletKit medium were incubated at 37°C and 5% CO_2_. After a confluence of about 80% was reached, the cells were either split or harvested by detaching the cells with 0.05% trypsin and 0.02% EDTA in PBS after washing with PBS. After centrifugation, the cells were taken up in medium and either seeded in new flasks or counted for experiments.

### 2.2 siPool transfection

Following cell numbers, per well of a six‐well culture plate was used for 96 hours transfection experiments: MelHo: 100,000 cells, MV3: 80,000 cells, fibroblasts: 120,000 cells, and WM1158: 140,000 cells. For 24‐h transfection experiments, 200,000 cells of each cell line, respectively, were used. The cells were transfected with 5 pmol siPool against PHF5A (siPHF5A) (siTools Biotech GmbH, Planegg, Germany) before attaching. SiPools were mixed with medium without phenol red and FCS and with 5 μL Lipofectamine RNAiMAX (Thermo Fisher Scientific, Waltham, Massachusetts, USA) per sample. The transfection mix was incubated for 20 min before pipetting to 1.5 mL cultivation medium. A siPool control (siCtrl) (siTools Biotech GmbH, Planegg, Germany) was used as negative control.

### 2.3 RNA isolation and cDNA synthesis

For isolation of total cellular RNA, the E.Z.N.A. Total RNA Kit I (Omega Bio‐Tek, Norcross, GA, USA) was used following the manufacturers protocol. Reverse transcription was performed with the Superscript II Reverse Transcriptase Kit (Invitrogen, Groningen, Netherlands), following the product manual. 500 ng of the isolated RNA were used for reverse transcription.

### 2.4 Quantitative real time‐PCR (qRT‐PCR)

To analyse the relative mRNA expression of different genes, qRT‐PCR was performed by using LightCycler® 480 II devices (Roche, Basel, Switzerland). The forward and reverse primers of each target gene and the housekeeping gene *β‐actin* were received from Sigma Aldrich. The PCR mixture consists of 25 ng cDNA template, 0.5 μM forward primer, 0.5 μM reverse Primer and 10 μL LightCycler® 480 SYBR Green I Master Mix (Roche, Basel, Switzerland) in a total volume of 20 μL. Following PCR program was performed: 10 min at 95°C, 45 cycles with 10 s at 95°C, 10 s at 60°C and 20 s at 72°C. For calculation, the ΔCP method was used.

### 2.5 Western blot protein analysis

The harvested cells were resuspended in 80 μL radio‐immunoprecipitation assay (RIPA) buffer (Roche, Basel, Switzerland) and incubated at 4°C for 15 min. After centrifuging at 13,000 rpm for 10 min, the concentration of the protein‐containing supernatant was determined by Pierce™ BCA protein assay (Thermo Fisher Scientific, Waltham, Massachusetts, USA). 40 μg of RIPA lysate were loaded on 12.75% gels and separated by SDS‐PAGE. The gels were blotted onto PVDF membranes (Bio‐Rad, Hercules, CA, USA) at 400 mA and 15 V for 1 h. To prevent unspecific bindings, the membranes were blocked with 5% non‐fat dried milk (NFDM) or 5% Bovine Serum Albumin (BSA) dissolved in TBS‐T for 1 h. Subsequently, the primary antibody (against PHF5A, ß‐actin, phospho‐eIF2α, eIF2α, ATF4 and ATF6 (full length)/ATF6‐N, respectively) was incubated over night at 4°C. Following antibodies and dilutions were used: anti‐PHF5A (from rabbit, 1:1000 in 5% NFDM/TBS‐T, Proteintech 15554‐1‐AP), anti‐β‐actin (from mouse, 1:5000 in PBS, Sigma Aldrich A5441), anti‐phospho‐eIF2α (from rabbit, 1:500 in 5% NFDM/TBS‐T, Abcam ab32157), anti‐ eIF2α (from mouse, 1:1000 in 5% NFDM/TBS‐T, Abcam ab5369), anti‐ATF4 (from rabbit, 1:1000 in 5% BSA/TBS‐T, Cell Signalling 11815), anti‐ATF6 (from mouse, 1:1000 in 5% NFDM/TBS‐T, Santa Cruz sc‐166659). After three washing steps with TBS‐T, the respective horseradish‐peroxidase‐coupled secondary antibody (1:2000 in TBS‐T, 7074 and 7076, Cell Signalling, Danvers, MA, USA) was applied for 1 h at room temperature. The Blots were incubated for three times again with TBS‐T, before visualization with Clarity™ Western ECL Substrate (Bio‐Rad, Hercules, California, USA) by chemiluminescence (Chemostar Imager, Intas, Goettingen, Germany). The signals were quantified using LabImage software (Kapelan Bio‐Imaging GbmH, Leipzig, Germany) with β‐actin (1:5000 in 5% BSA, Sigma‐Aldrich, A5441) as housekeeping protein.

### 2.6 Cell proliferation assay

For quantification of viability and proliferation of the cells, the XTT method (cell proliferation kit II, Roche, Basel, Switzerland) was used. For this, 200,000 cells per six‐well were transfected with the particular siPool. After 24 h, cells were seeded as triplicates in 96 wells (500 cells/well), cultivated in 100 μL of the respective medium without phenol red for 6 days. Measurement occurred every day except of days two and three, starting one day after seeding. The XTT reagent was used according to the manufacturer's instructions and the absorbance at 490 nm was measured with a CLARIOStar plate reader (BMG Labtech GmbH, Ortenberg, Germany) 4 h after adding the XTT reagent.

### 2.7 Clonogenic assay

Twenty‐four hours after transfection with siPHF5A and siCtrl, respectively, 200 cells were seeded in a well of a six‐well culture plate and cultivated for 7 days to allow colony formation. Subsequently, the colonies were fixed and stained for 30 min at room temperature, using 6% glutaraldehyde and 0.36% crystal violet solution. For calculation, the number of formed colonies was detected by CellSens Dimension software (Olympus K.K., Shinjuku, Tokyo, Japan).

### 2.8 Apoptosis FACS

For quantification of the number of apoptotic cells, the treated cell line (96 h, siPHF5A and siCtrl, respectively) was harvested as described before. The cultivation medium was collected too, which contained already detached cells. The cells were washed with PBS and stained using Annexin V‐FITC Apoptosis Kit (MyBioSource, San Diego, CA, USA) following the manufacturer's protocol. The analysis was performed by flow cytometry with BD LSRFortessa™ Cell Analyser (BD Biosciences, Franklin Lakes, NJ, USA) and interpretation by BD FACSDiva 9.0 software (BD Biosciences, Franklin Lakes, NJ, USA).

### 2.9 Collagen contraction assay

To mimic the effect of wound healing in fibroblasts, 250,000 cells were seeded in low‐attachment‐plates (Corning, CLS3471, New York, USA). For that, 250 μL cells were mixed with 1.5 mL 1.5‐fold DMEM medium (low glucose, without phenol red) and 250 μL collagen (Rat Tail Collagen Type 1, BD Biosciences, Franklin Lakes, NJ, USA). During incubation at 37°C and 8% CO_2_, it was possible to measure the diameter of the collagen ring at different time points for quantification. The cells were observed until they contracted completely.

### 2.10 Splicing analysis by MAJIQ

The used RNA‐seq data of MelHo and MV3 after the knockdown of PHF5A relative to siCtrl were prepared as described previously^21^ (BioProject ID PRJNA1044935).

Differential splicing analysis was performed using MAJIQ v.2.4 using the RNA‐seq data of all biological replicates.^22^ We used the GENCODE release 24 for the gene annotation within MAJIQ.^23^ The two cell lines MelHo and MV3 were analysed separately. First, MAJIQ's build function was run to build a splice graph. Then the deltapsi function was run to call differentially spliced junctions by contrasting the control and siPHF5A group within each cell line. The reported differentially spliced junctions were filtered based on the change of splicing in percent, *Δ*𝜓 ≥ 0.3, and the changing probability ≥0.95 in order to be considered as significant.

Motif discovery analysis was performed using STREME v.5.5.4 with default parameters in a discriminative mode. Motifs with a *p*‐value ≤ 0.5 are considered significant.^24^ From each differentially spliced event, two sequences of length 100 are generated in upstream and downstream, i.e. (start 2212100, start) and (end, end +100). We performed the analysis for 3 different *Δ*𝜓_c_ cutoffs: 0.3, 0.4, 0.5. For each *Δ*𝜓_c_, we created the following three groups based on the observed *Δ*𝜓_o_:

- More skipped: Sequences with *Δ*𝜓_o_ < −*Δ*𝜓_c_ are assigned to this group, showing exons that are skipped more in siPHF5A compared to control.
- More included: Sequences with *Δ*𝜓_o_ > *Δ*𝜓_c_ are assigned to this group, representing exons that are more included in siPHF5A compared to control.
- Control: the remaining sequences (−*Δ*𝜓_c_ < = *Δ*𝜓_o_ < = *Δ*𝜓_c_) are assigned to this group.

To detect enriched motifs, STREME was then run two times by contrasting the groups (i) More skipped with Control and (ii) More included with Control.

### 2.11 Thioflavin T staining

30,000 transfected cells were seeded in 12‐wells on a coverslip until the cells attach. After that, the coverslips were washed with PBS two times and the cells fixated with 4% PFA per coverslip. After 20 min of incubation at room temperature, the cells were washed with PBS again and incubated in 0.5% Thioflavin T (catalogue no. 211760050, Thermo Fisher Scientific, Waltham, Massachusetts, USA) in 0.1 M HCl solution for 20 min in the dark. After washing the cells with PBS for three times and with double distilled water one time, the coverslips were mounted with Aqua Polymount (Polysciences) and analysed on an IX83 microscope (Olympus). For the evaluation, the proportion of cells showing protein accumulations was determined by manual counting. Second, ImageJ software was used to determine the average size of the visible accumulations in positive cells. For this, 5 cells per replication and per treatment were used.

### 2.12 Immunohistochemical staining

The immunohistochemical staining of the patient‐derived human malignant melanoma samples was performed as described previously.^25^ For this analysis, 10 primary tumour samples and 10 metastasis samples were stained. The tissue collection is located at the Institute of Pathology (University of Regensburg, Germany). The PHF5A antibody (15554‐1‐AP, Proteintech, Planegg‐Martinsried, Germany) was used in a dilution of 1:50. The sampling and handling of patient material was performed in accordance with the ethical principles of the Declaration of Helsinki. The use of human tissue material was authorized by the local ethics committee of the University of Regensburg (application numbers 09/11 and 03/151).

### 2.13 Bioinformatics

Patient survival rates according to PHF5A expression were analysed by applying the TCGA‐derived datasets published by the Protein Atlas Database (retrieved on November 10, 2023; https://www.proteinatlas.org/ENSG00000100410-PHF5A/pathology/melanoma). GO term enrichment analysis using the STRING database was performed as described previously.^26^ For further analysis of PHF5A expression Mexpress database (https://mexpress.ugent.be), GEPIA database (http://gepia.cancer-pku.cn/index.html) and COSMIC database (https://cancer.sanger.ac.uk/cosmic) were used, each retrieved on June 11, 2024.

### 2.14 Statistical analysis

Each experiment was performed with at least three biological replicates. Statistical figures and tests were created by using GraphPad Prism 10 (Version 10.0.1, GraphPad Software Inc., San Diego, CA, USA). Comparisons between the two treatment groups were analysed by the Student's unpaired *t*‐test. For analysing more than two groups, one‐way or two‐way analyses of variance (ANOVA, Bonferroni multiple comparison test) were used. Column graphs are shown as mean ± SEM (range) or percent. *p*‐Values less than 0.05 were determined as statistically significant. If not otherwise stated, tests were not significant.

## 3 RESULTS

### 3.1 PHF5A is upregulated in most melanoma cell lines

**FIGURE 1:**
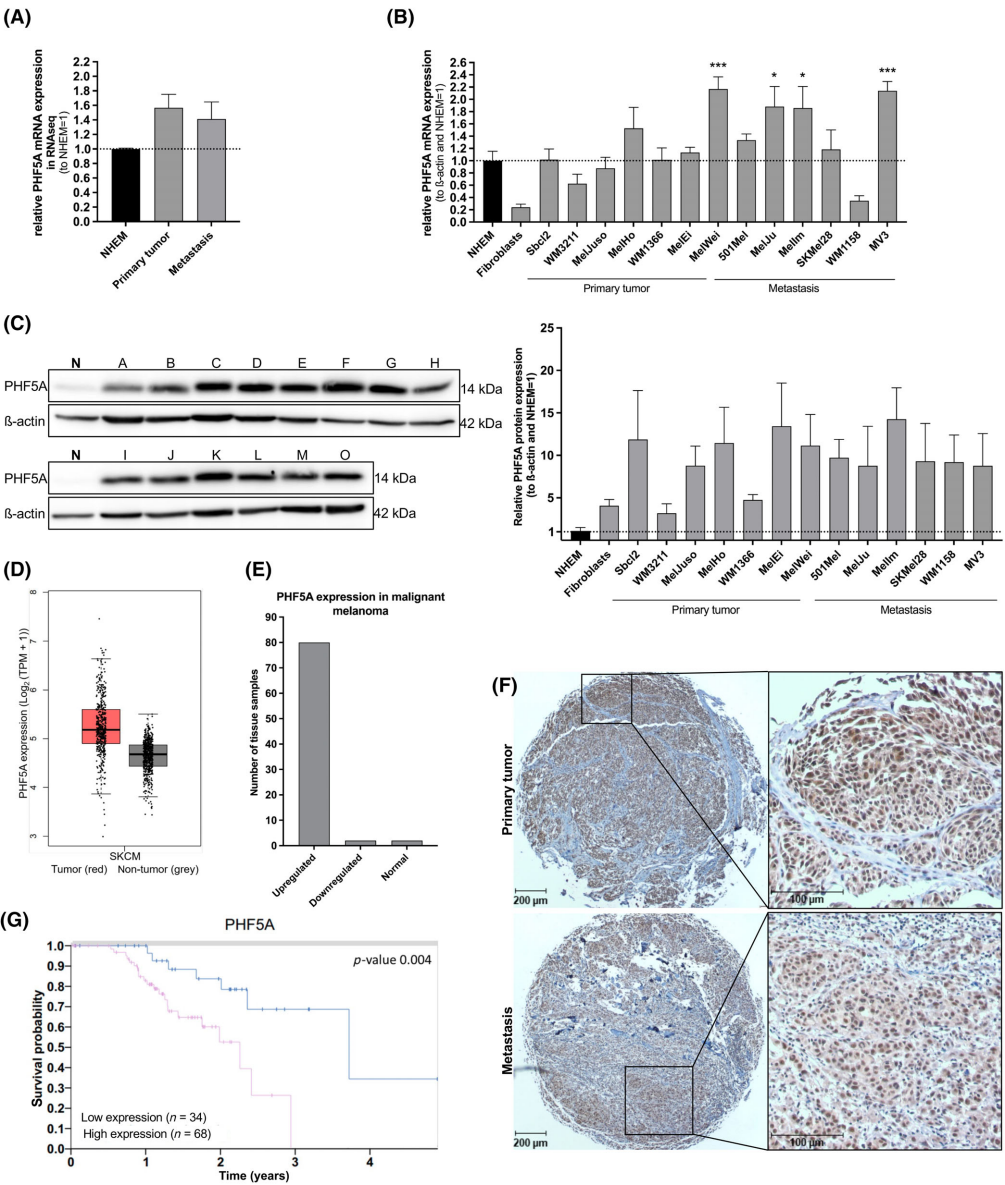
PHF5A is upregulated in malignant melanoma cell lines and correlates with poor survival. (A) Relative PHF5A mRNA levels of primary tumour and metastatic cell lines relative to expression in NHEM cells, (2 NHEM, 8 primary tumour and 4 metastatic cell lines were used). Used data were taken from RNA‐sequencing data.^26^ (B) Relative PHF5A mRNA levels normalized to β‐actin and NHEM mRNA expression, measured with qRT‐PCR in different melanoma cell lines (*n* = 3 (WM3211, MelJuso, MelHo, WM1366, MelEi, 501Mel, MelJu, MelIm, SKMel28, WM1158), *n* = 4 (Sbcl2, MelWei), *n* = 5 (MV3), *n* = 11 (NHEM) and Fibroblasts (*n* = 3)). (C) Relative PHF5A protein levels (Western blot analysis) in melanoma cell lines (*n* = 3 (I: 501Mel), *n* = 4 (D: MelJuso, E: MelHo), *n* = 5 (C: WM3211, H: MelWei, J: MelJu), *n* = 7 (G: MelEi, L: SKMel28, O: MV3), *n*= 8 (M: WM1158), *n* = 11 (F: WM1366), *n* = 15 (K: MelIm, B: Sbcl2)) and A: Fibroblasts (*n* = 5) compared to N: NHEM (*n* = 5); β‐actin was used as a housekeeping protein. One representative Western blot is shown. (A–C) Mean ± SEM, *p < 0.05, ***p < 0.001, One‐way ANOVA and subsequent Bonferroni Multiple Comparison Test, line at *y* = 1.0. (D) Analysis of PHF5A expression in skin cutaneous melanoma (SKCM) samples (red) compared to non‐tumour samples (grey). Data obtained from GEPIA database, *p*‐value cut‐off = 0.01, |Log_2_FC| Cut‐off = 1, retrieved on June 07, 2024. (E) Number of tumour tissue samples depending on their PHF5A regulation. Data obtained from COSMIC database, upregulation: *Z*‐Score >2.0, downregulation: *Z*‐Score < −2.0, normal: −2.0 < *Z*‐Score <2.0; retrieved on June 07, 2024. (F) Immunohistochemical staining of PHF5A protein (exemplary images) of human patient derived tumour samples from malignant melanoma primary tumour (upper panel) and metastasis (lower panel). Stained with PHF5A antibody in dilution of 1:50. (G) Kaplan–Meier plot depicting survival probability of melanoma patients. Dataset obtained from “The Human Protein Atlas” Database. Differentiated by low (red) or high (blue) expression of PHF5A, retrieved on November 10, 2023.

To investigate the PHF5A expression in malignant melanoma, we analysed existing RNA‐seq data^26^ and found an upregulation of *PHF5A* mRNA in primary tumour cell lines, as well as in metastasis derived cell lines compared to normal human epidermal melanocytes (NHEMs, Figure 1A). To confirm these findings, we performed qRT‐PCR and Western Blot analysis, which showed an upregulation of PHF5A expression on mRNA and an even higher expression on protein levels in most melanoma cell lines. Fibroblasts showed less mRNA expression, however a higher protein expression of PHF5A in comparison to NHEM (Figure 1B,C). To further verify these observations, we also interpreted data sets from other databases. For example, GEPIA data confirm that higher PHF5A expression can be detected in skin cutaneous melanoma (SKCM) samples compared to non‐tumour samples (Figure 1D). Supporting these findings, the COSMIC database shows that in all tissue samples for which regulation of expression is indicated (*n* = 84), the majority (80 samples) show upregulation (Figure 1E). These in vitro data were supported by immunohistochemical staining of PHF5A in patient‐derived human tissue samples of 10 primary melanomas and 10 metastases, which all revealed a high homogeneous expression of PHF5A in the melanoma samples, especially in the cell nuclei (Figure 1F, Figure S2). The staining occurred in all tumour cells, again confirming the high PHF5A expression in melanoma cells. Data from the Mexpress database confirm these observations, as no correlation between PHF5A expression and the melanoma Clark level or the Breslow depth level can be detected here either. To further assess the importance of PHF5A in malignant melanoma, we correlated its expression profile with the survival probability of melanoma patients by applying the TCGA‐derived datasets published by the Protein Atlas Database. The survival probability is significantly decreased in the case of a high PHF5A expression (*p* = 0.004) (Figure 1G, data obtained from “The Human Protein Atlas”, retrieved on November 10, 2023). Supporting these results, a correlation between high PHF5A expression and poor overall survival can also be drawn with data of the Mexpress database (*p* < 0.001), indicating that PHF5A might contribute to tumourigenesis and maintenance of malignant melanoma. PHF5A expression also appears to play a role in the response to treatment. For example, treatment with vemurafenib, inhibiting activity of mutated BRAF, leads to a downregulation of PHF5A in melanoma cells.^27^ This suggests that PHF5A is closely involved in the ERK signalling pathway, which plays a role in the development of tumours. All these results reflect the essential biological role that PHF5A has in malignant melanoma.

### 3.2 PHF5A knockdown inhibits proliferation and promotes apoptosis in melanoma cell lines

**FIGURE 2:**
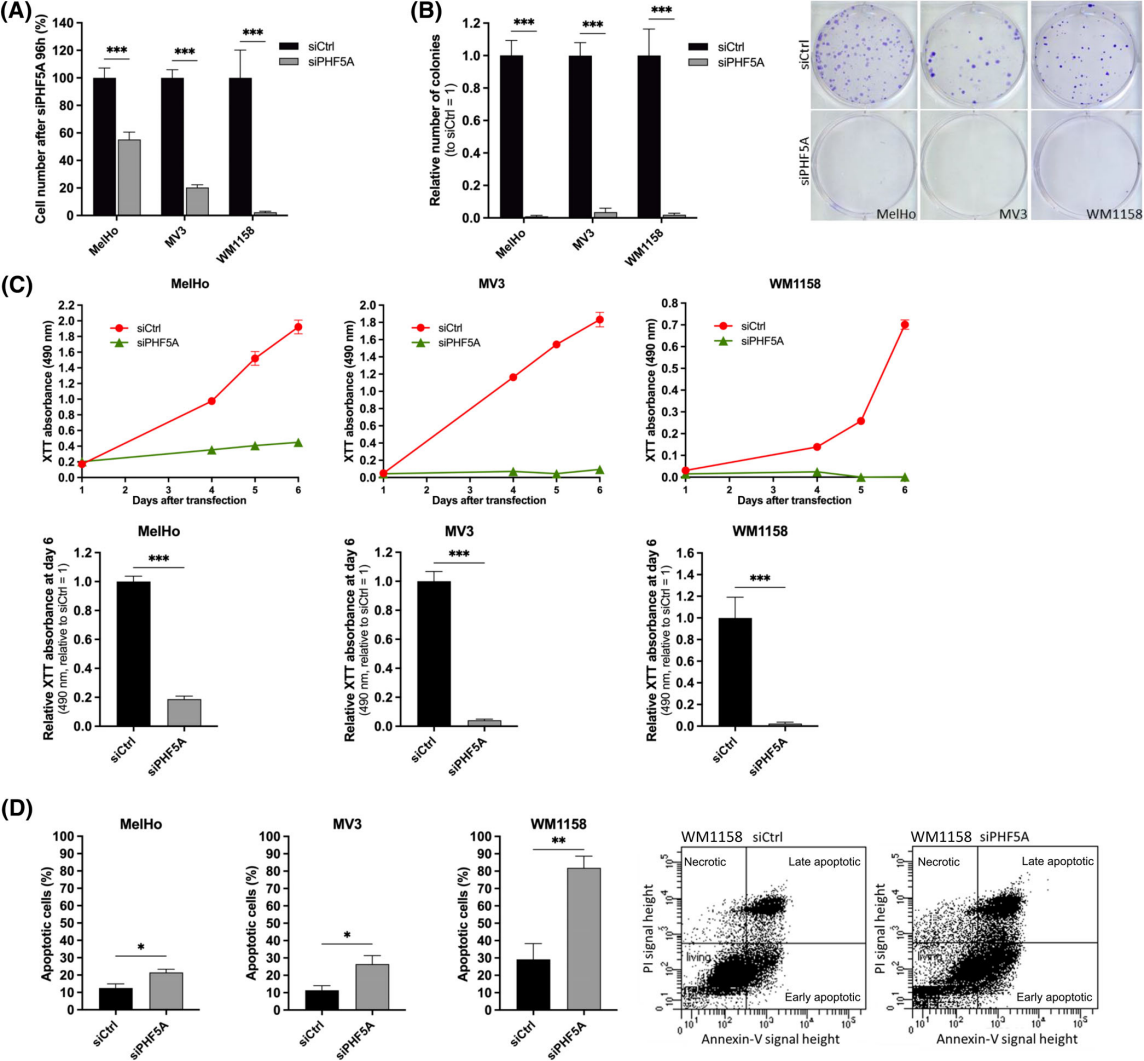
Knockdown of PHF5A significantly inhibits cell proliferation and increases apoptosis rate in malignant melanoma cells. (A) Cell number of indicated melanoma cell lines 96 h after transfection with siPHF5A relative to siCtrl in percent (*n* = 3 (WM1158), *n* = 4 (MV3), *n* = 8 (MelHo) (mean ± SEM, **p < 0.01, ***p < 0.001, Two‐way ANOVA and subsequent Bonferroni Multiple Comparison Test)). (B) Clonogenic assay of indicated melanoma cell lines after transfection with siPHF5A. Bar graph shows the number of colonies normalized to siCtrl (*n* = 5 (WM1158), *n* = 8 (MelHo), *n* = 10 (MV3) (mean ± SEM, ***p < 0.001, Two‐way ANOVA and sub‐sequent Bonferroni Multiple Comparison Test)). One exemplary picture per cell line is shown. (C) XTT viability assay of indicated cell lines treated with siCtrl or siPHF5A, respectively, for 24 h before the assay. The XTT absorbance at 490 nm is shown at presented time points. For each cell line, one representative graph is shown. Assay was performed with three technical replicates per sample. Mean of XTT absorbance at day 6 relative to siCtrl (*n* = 3 (MV3), *n* = 4 (MelHo), *n* = 7 (WM1158) (mean ± SEM, *p < 0.05, ***p < 0.001, one‐sample *t*‐test)). (D) Quantification of apoptotic cells 96 hours after knockdown of PHF5A in indicated cell lines compared to cells transfected with siCtrl, respectively. Cells were stained with Propidium Iodid (PI) and Annexin V‐FITC and analysed by flow cytometry. Representative plots of flow cytometric detection of apoptotic cells exemplarily shown for WM1158. Bar graphs show the summary of early and late apoptotic cells in percentage (*n* = 4 (MelHo, MV3, WM1158) (mean ± SEM, *p < 0.05, **p < 0.01, one‐sample *t*‐test)).

To investigate the importance of PHF5A for melanoma development, we performed siPool‐mediated knockdown of PHF5A in the three melanoma cell lines MelHo, MV3 and WM1158, and achieved a significant reduction of PHF5A expression on mRNA and protein level (Figure S1A,B). The knockdown severely impacted cell proliferation, leading to significantly decreased cell counts after 96 h of PHF5A knockdown in all samples (Figure 2A). Of all cell lines, WM1158 was affected the most with a reduction in cell number of about 97% after the knockdown of PHF5A. To further analyse the effect of a PHF5A knockdown on melanoma cell tumourigenicity, we performed a clonogenic assay (Figure 2B). The knockdown resulted in a loss of clonogenicity as indicated by a significantly decreased number of colonies. We further performed XTT proliferation assays, starting 24 h after transfection with siPHF5A (Figure 2C). Here, we could reveal a complete inhibition of proliferation in MV3 and WM1158 and a strongly reduced proliferation in MelHo in siPHF5A transfected cells compared to controls. Because of the strong effects in all cell lines, we hypothesized that the knockdown of PHF5A induces cell death. To investigate this, we quantified apoptotic cells in MelHo, MV3, and WM1158 cell lines 96 h after the knockdown of PHF5A using propidium iodide/Annexin‐V‐staining and analysed them via flow cytometry. From this, we observed significantly increased apoptosis in all the cell lines (Figure 2D). As seen in the cell number and proliferation assay before, WM1158 were most sensitive to PHF5A knockdown, which led to approximately 80% of cells being apoptotic.

### 3.3 Fibroblasts are resistant to the knockdown of PHF5A

**FIGURE 3:**
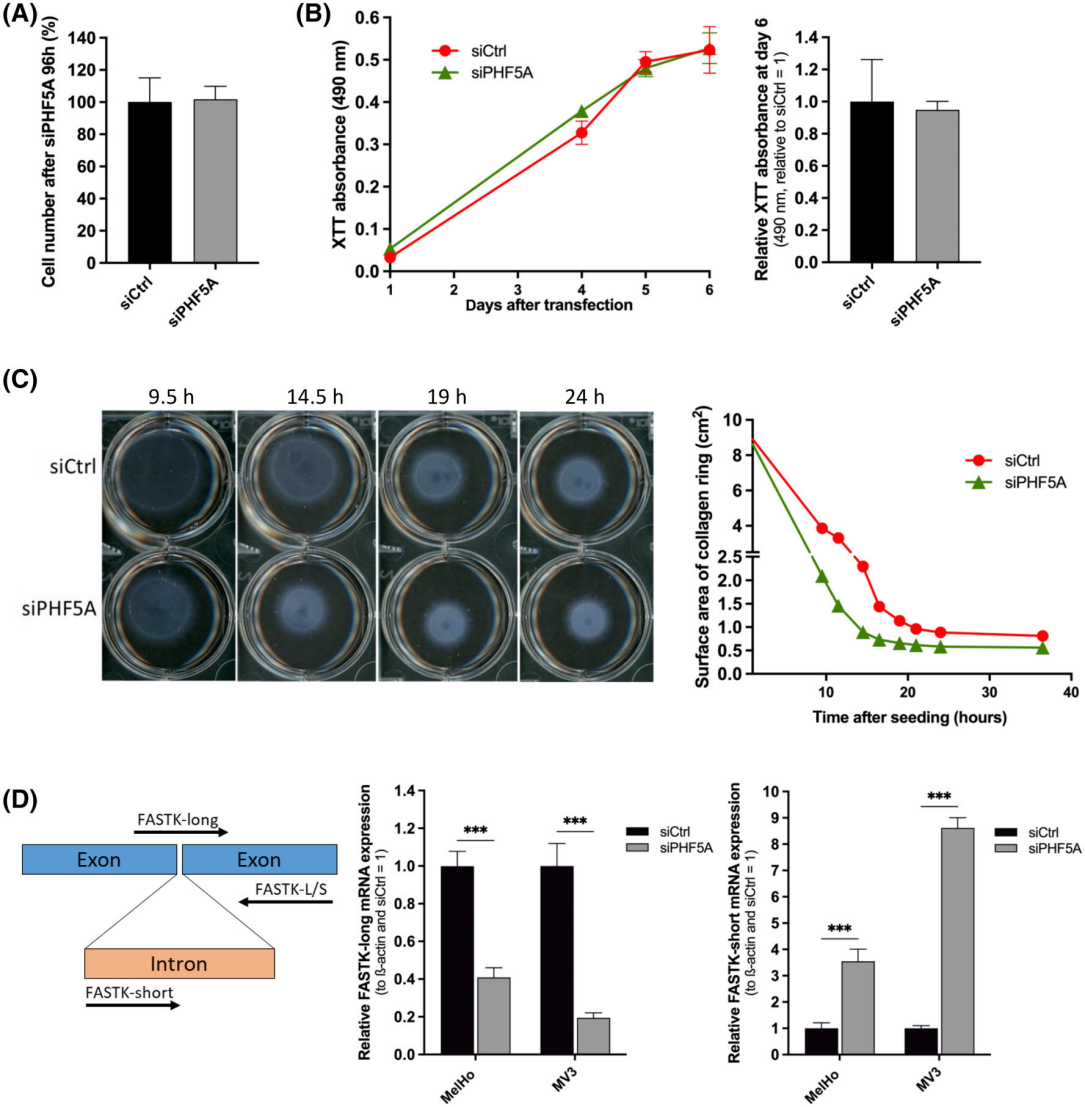
Knockdown of PHF5A does not affect viability and ability of contraction in fibroblasts. (A) Cell number of fibroblasts 96 h after transfection with siPHF5A relative to siCtrl in percent (*n* = 4, mean ± SEM). (B) XTT viability assay of fibroblasts after treatment with siCtrl or siPHF5A, respectively, for 24 h before the assay. XTT absorbance at 490 nm is shown at presented time points. One representative graph is displayed, performed with three technical replicates per sample. The bar graph shows the mean of XTT absorbance shown in B at day 6 relative to siCtrl (*n* = 4, mean ± SEM). (C) Collagen contraction assay was performed 96 hours after transfection with siCtrl or siPHF5A, respectively, in fibroblasts. An exemplary picture of the contraction of the fibroblast/collagen mix at indicated time points is displayed. The graph shows a representative development of the surface area of the visible collagen ring (cm^2^) after siPHF5A compared to siCtrl over time. (D) Graphical scheme on the left side shows primer binding positions, which demonstrate FASTK‐short as product in case of intron retention and FASTK‐long in case of correct splicing. Relative mRNA expression of splicing variant FASTK‐long and FASTK‐short after treatment with siPHF5A compared to siCtrl in melanoma cell lines MelHo and MV3 analysed by qRT‐PCR (*n* = 4 (MV3), *n* = 6 (MelHo) (mean ± SEM, ***p < 0.001, Two‐way ANOVA and subsequent Bonferroni Multiple Comparison Test)).

To prove the potential of PHF5A as a therapeutic target, we investigated the impact of its knockdown on normal epidermal cells like fibroblasts, realized with siPHF5A and siCtrl after 96 h again (Figure S1C,D). The knockdown did not lead to a significant decrease in cell number (Figure 3A). Analysis of proliferation performing the XTT assay also did not show any negative impact of PHF5A knockdown compared to control (Figure 3B). An important function of fibroblasts in human tissues is their role in wound healing. After transfecting fibroblasts with siPHF5A for 96 h, the cells were still able to contract collagen, indicating that the knockdown did not have functional consequences on the cells (Figure 3C).

### 3.4 PHF5A knockdown leads to alternative splicing events in melanoma cell lines

To analyse the molecular mechanism leading to apoptosis of melanoma cells after PHF5A knockdown and since the function of PHF5A during the process of splicing is known,^28^, ^29^ we first focused on the occurrence of alternative splicing events of the antiapoptotic gene *FASTK*, which was already described in breast cancer.^19^ In this cancer type, the loss of PHF5A led to the retention of intron 5 in *FASTK*, resulting in a non‐functional protein (FASTK‐short, Figure 3D). Specifically, the intron retention causes a frame shift creating a premature stop codon. By performing qRT‐PCR after transfection with siPHF5A for 96 hours, we observed a reduction of the functional form of *FASTK* (FASTK‐long) as well as an increased expression of the non‐functional, alternative splicing variant FASTK‐short in MelHo and MV3 melanoma cells. Due to severe cell loss, we were not able to perform the experiment in the cell line WM1158.

**FIGURE 4:**
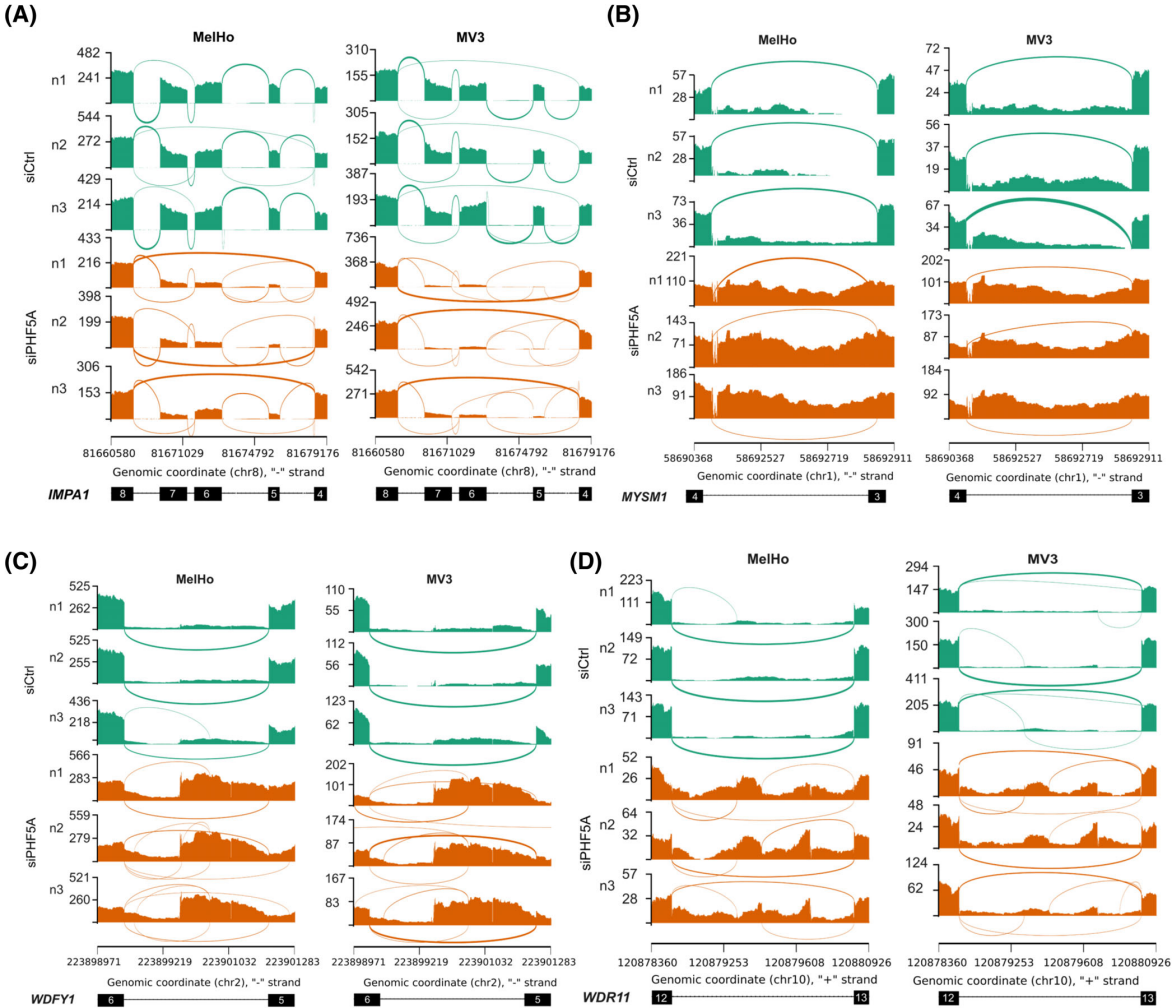
Examples of differentially spliced genes after siPHF5A treatment. (A–D) Sashimi plots showing RNA‐seq read coverage (*y* axis) and the usage of an exon‐exon connection (thickness of line) using IGV for the cell lines MelHo (left) and MV3 (right) treated with siPHF5A (orange) and without (green) for all three biological replicates. The genomic coordinates are given on the *x* axis together with the reference annotation of the respective gene. (A) Depiction of alternative splicing in *IMPA1*. (B) Depiction of intron retention in *MYSM1*. (C) Depiction of partial intron retention in *WDFY1*. (D) Depiction of intron retention in *WDR11*.

**FIGURE 5:**
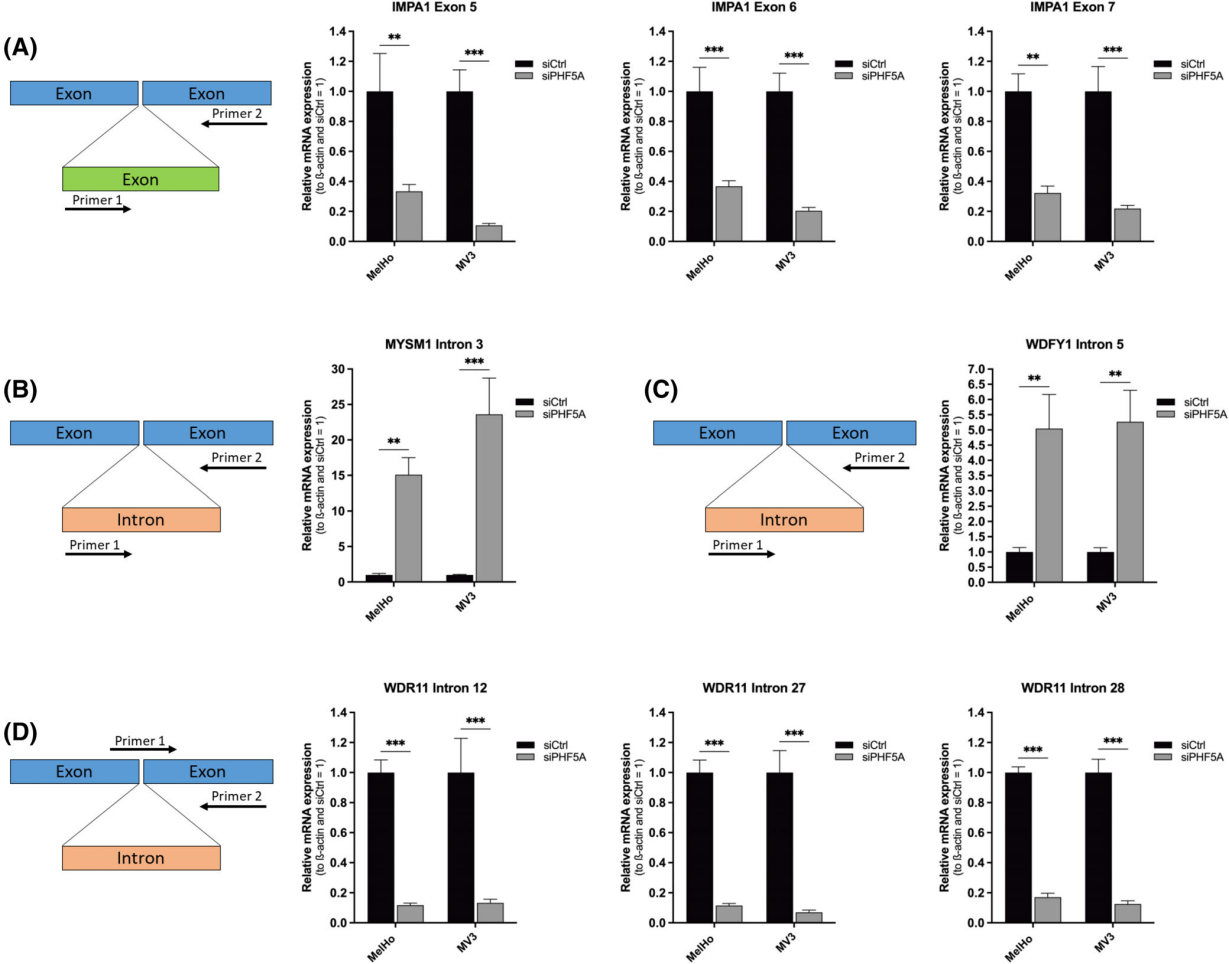
Knockdown of PHF5A leads to abnormal splicing events of several tumour‐relevant genes in melanoma cells. (A–D) Graphical schemes on the left show primer binding positions, which describe the observed PCR product. Relative mRNA expression of respective splicing variant after treatment with siPHF5A compared to siCtrl, respectively, in melanoma cell lines MelHo and MV3 analysed by qRT‐PCR (at least four biological replicates) (mean ± SEM, **p < 0.01, ***p < 0.001, Two‐way ANOVA and subsequent Bonferroni Multiple Comparison Test).

Given that PHF5A is an important splicing factor, we hypothesized that its downregulation could have, besides the gene *FASTK*, a broad impact in general on splicing. To define the effect of siPHF5A treatment on transcriptome‐wide splicing, we performed a differential splicing analysis with MAJIQ^22^ on RNA‐seq data of MelHo and MV3 after siPHF5A transfection. The two cell lines were analysed separately for differences between the control and siPHF5A groups. The results were filtered by the change of splicing in percent spliced‐in, *Δ*𝜓 ≥ 30%, and the changing probability ≥0.95 resulting in 647 and 1949 differentially spliced genes in MelHo and MV3, respectively (File S1). Remarkably, only 425 genes were found in both cell lines (File S3), which could be due to differences in splicing but is more likely due to differences in gene expression, e.g. genes linked to pigmentation like DCT, which are only expressed in MelHo but not in MV3. We concentrated on common regulation in both cell lines and revealed similar changes in splicing. For example, for the tumour‐promoting genes *IMPA1, MYSM1, WDFY1* and *WDR11* (Figure 4).^30^, ^31^, ^32^, ^33^, ^34^, ^35^, ^36^, ^37^ Using qRT‐PCR, we were able to confirm a significantly higher rate of exon skipping in *IMPA1* (Figure 5A). Significant intron retention was visible in *MYSM1*, the gene encoding for a metalloprotease (Figure 5B), and *WDFY1*, a gene of WD repeat and FYVE domain‐containing protein (Figure 5C), which plays an important role in tumour development. Most intron retentions were found in *WDR11*, which showed significant events in intron 12, 27, and 28 in MelHo as well as MV3 (Figure 5D).

Based on the differentially spliced genes, we further performed GO term enrichment analysis using the STRING database.^38^, ^39^ The resulting high‐ranked terms of the analysis were related to splicing (Uniprot Keyword, KW‐0025), metabolic processes (e.g. GO:0043170), cellular transport (GO:0046907), and localization (e.g. GO:0033036) among others (File S3).

In addition, a motif enrichment analysis was performed to investigate whether there was a homogeneous pattern that caused exons to be either more or less skipped in siPHF5A compared to the control group. The analyses with STREME^24^ based on the *Δ*𝜓 values did not reveal any motif explaining the splicing defects after siPHF5A treatment.

### 3.5 Knockdown of PHF5A induces UPR and alters expression of apoptosis‐related genes

**FIGURE 6:**
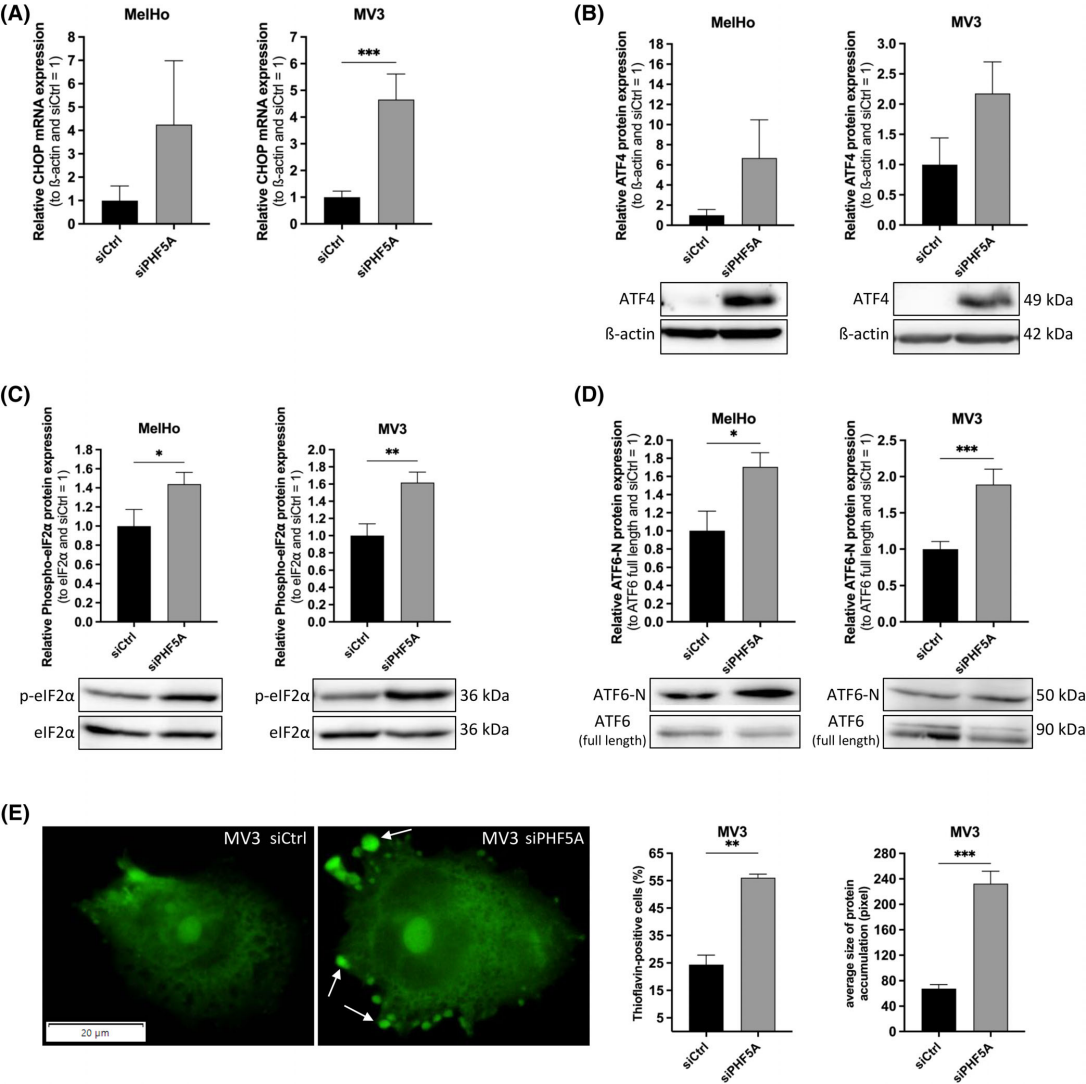
Knockdown of PHF5A leads to induction of unfolded protein response (UPR). (A) Relative CHOP mRNA expression to β‐actin and siCtrl, measured with qRT‐PCR in MelHo and MV3 (*n* = 10 (MelHo), *n* = 15 (MV3) (mean ± SEM, ***p < 0.001, one‐sample *t*‐test compared to 1)). (B) Relative ATF4 protein expression after siPHF5A treatment and siCtrl, respectively, in MelHo and MV3, analysed by Western Blot (*n* = 8 (MelHo), *n* = 11 (MV3) (mean ± SEM, one‐sample *t*‐test)). One representative blot per cell line is shown. β‐actin was used as a housekeeping protein. (C) Phosphorylated eIF2α protein expression in comparison to eIF2α and siCtrl in MelHo and MV3, analysed by Western Blot (*n* = 18 (MV3, MelHo) (mean ± SEM, *p < 0.05, one‐sample *t*‐test compared to 1)). One representative blot per cell line is shown. (D) Activated ATF6‐N protein expression in comparison to full length ATF6 and siCtrl in MelHo and MV3, analysed by Western Blot analysis (*n* = 14 (MV3), *n* = 19 (MelHo) (mean ± SEM, *p < 0.05, **p < 0.01, one‐sample *t*‐test)). One representative Western blot per cell line is shown. (E) Thioflavin T staining of MV3 96 h after transfection with siCtrl or siPHF5A, respectively, for labeling protein aggregates. Loss of PHF5A leads to accumulation of protein aggregates, visible as green dots, which are marked with arrows. Percentage of cells showing protein accumulations (Thioflavin‐positive). Average size of protein accumulations in Thioflavin‐positive cells, 5 cells per replicate were analysed (*n* = 3 (mean ± SEM, **p < 0.01, ***p < 0.001, one‐sample *t*‐test)).

Besides focusing on alternative splicing, we investigated a potential involvement of the unfolded protein response (UPR), since defects in splicing might result in protein misfolding and UPR‐induced apoptosis. Here, we analysed two of the relevant signalling pathways: PKR‐like endoplasmic reticulum kinase (PERK) and Activating transcription factor 6 (ATF6). The expression of PERKs major effector protein, the pro‐apoptotic C/EBP homologous protein (CHOP) transcription factor, was assessed on mRNA level to evaluate this hypothesis. We found significant upregulation of *CHOP* on mRNA level after transfection of siPHF5A for 96 hours, indicating that knockdown of PHF5A causes UPR activation in melanoma cells, potentially further explaining the observed apoptotic cell death (Figure 6A). To pursue this hypothesis, we analysed the expression of further players of UPR on the protein level. Western Blot analysis revealed an upregulation of the upstream signal molecule Activating transcription factor 4 (ATF4) (Figure 6B). To activate ATF4, phosphorylation of serine 51 of the eukaryotic translation initiation factor 2α (eIF2α) is necessary,^40^, ^41^ which we could confirm by western blot analysis (Figure 6C). Besides, in case unfolded proteins accumulate, another opportunity is that ATF6 is cleaved to activate UPR downstream target genes.^42^, ^43^ We could show an increased amount of cleaved ATF6 (ATF6‐N) as well as a reduction of full‐length ATF6 on protein level after knockdown of PHF5A, indicating that a loss of PHF5A in melanoma cells activates also this second UPR pathway (Figure 6D). To further support our findings, we performed Thioflavin T staining for labelling protein accumulations (Figure 6E). In MV3, we were able to detect accumulated proteins in the cytoplasm, marked with arrows, which indicate misfolded proteins. After a loss of PHF5A, we observed a significantly increased number of cells showing these accumulations. Furthermore, the size of the protein clusters was significantly larger in siPHF5A‐treated cells, which also indicates a higher degree of accumulation.

## 4 DISCUSSION

In this study, we investigated the role of PHD finger protein 5A (PHF5A) in malignant melanoma. PHF5A is a component of the SF3B splicing complex and is involved in pre‐mRNA splicing by branch point recognition.^13^ As a key mechanism of transcriptional control and regulation, RNA splicing is necessary for many biological processes. While alternative splicing enables cells to create more diversity in their transcriptomes,^44^ its dysregulation can lead to the formation of cancer and other diseases.^45^, ^46^, ^47^ Abnormal splicing patterns can help tumours become resistant to therapy or less prone to apoptosis. Most mechanisms of splicing as well as functions of several parts of the spliceosome are still unknown. New insights in tumour‐promoting splicing events or responsible proteins would be a key step in discovering new molecular targets for cancer therapeutics.

Here, we could show that PHF5A is upregulated in malignant melanoma cells compared to normal human epidermal melanocytes (NHEM) and shows high protein expression in patient‐derived material. Additionally, high expression of PHF5A leads to worsened survival prognosis for melanoma patients. This enhanced expression pattern of PHF5A and similar survival prognosis has also been described for other cancer entities, highlighting *PHF5A* as a critical gene for cancer progression.^48^, ^49^, ^50^ An upregulation of splicing factors like PHF5A can increase the number of splicing events, which is beneficial for high proliferative tumours. For example, Gout and colleagues revealed that the overexpression of serine/arginine‐rich splicing factor 1 (SRSF1) correlates with a more aggressive phenotype of Non‐Small Cell Lung Carcinoma (NSCLC) cells.^51^ Previous studies showed similar effects for the splicing factors BUD31 and SRSF1, the knockdown of which leads to inhibited proliferation and increased apoptosis in other cancer types.^11^, ^52^ These findings show the indispensability of splicing factors for the maintenance of different tumour types, highlighting the essential role of the splicing process for cancer entities. It is also shown that specific splicing variants only occur in cancer cells but not in non‐tumour tissues like a variant of epidermal growth factor receptor (EGFR), which does not contain exon 4.^53^ This leads to the translation of a permanently active protein, which promotes proliferation and thereby enhances the progression of different cancer types like gliomas, ovarian or prostate cancers. Further studies could link the upregulation of PHF5A to increased progression of cancer entities, such as glioblastoma multiforme or colorectal cancer, because of inhibited splicing,^20^, ^50^ supporting our findings.

In this study, we found that the loss of PHF5A leads to massive splicing defects in different target genes in malignant melanoma. We observed exon skipping as well as intron retention events in different genes after a knockdown of PHF5A in malignant melanoma cell lines. For example, we observed increased intron retention in the genes *MYSM1, WDFY1*, and *WDR11* after siPHF5A. The protein Myb‐Like SWIRM And MPN Domain‐Containing Protein 1 (MYSM1) is known to be responsible for deubiquitination of histone H2A but is shown to play an important role in malignant melanoma, too. Wilms and team revealed that MYSM1 is involved in the regulation of survival‐ or proliferation‐relevant genes, like *c‐MET*, leading to the progression of malignant melanoma.^31^ The same effects apply to WD repeat‐containing protein 1 (WDR11), which was shown to be involved in the Hedgehog signalling pathway and ciliogenesis.^36^ This pathway is closely related to different cancer types, including malignant melanoma. Recent studies revealed that the Hedgehog signalling pathway is abnormally activated in melanoma cells.^37^ After inhibitory experiments against the pathway, Peng and team were able to reach an inhibition of cell proliferation, migration, and invasion, as well as an increase of apoptotic events. Interestingly, there are also hints in the literature that WDR11 acts as a tumour suppressor, since it is disrupted in gliomas.^54^ The third intron retention candidate, WD Repeat And FYVE Domain Containing 1 (WDFY1) enhances the production of inflammatory factors or IFN‐β by positive regulation of TLR3‐ and TLR4 signalling pathways and activation of NF‐κB,^32^ thereby promoting progression of melanoma,^33^, ^34^ and other serious types of cancer.^35^ In this study, we have now identified PHF5A as a central regulator of WDR11. As demonstrated by *Inositol monophosphatase 1* (*IMPA1*), the knockdown of PHF5A can also cause exon skipping. IMPA1 is a very important player in the metabolism of inositol and shown to be upregulated in Triple‐negative breast cancer (TNBC), as well as a positive regulator of colony formation and proliferation of TNBC cells.^30^ In our study, both events, intron retention and exon skipping, resulted in frame shifts, which lead to the occurrence of a premature stop codon and subsequently presumably a non‐functional protein. Because of this, we assumed that correct and increased splicing of these genes is an essential step in melanoma progression.

Interestingly, we were not able to detect changes in splicing of every gene, although a PHF5A knockdown of about −95% was reached in our experimental setup. While a huge impact on many tumour‐relevant genes is visible, there are also parts of genes or entire genes, which were spliced correctly after the loss of PHF5A. This might be a common effect, which could be explained by the different GC content between introns and surrounding exons, which has been shown by Teng et al.^13^ Depending on the GC composition, it may be more difficult for the splicing machinery to recognize specific splicing sites. However, we were able to show that the effects, occurring after a loss of PHF5A, seem to be tumour‐specific, since the proliferation of fibroblasts and their ability to contract to collagen were not affected. As part of the connective tissue, fibroblasts are shown to play a crucial role in wound healing, since their loss or inactivity is linked to significantly decreased healing rates in ulcer experiments.^55^, ^56^ Since the knockdown of PHF5A does not affect the viability of these indispensable cells of the human skin but results in apoptosis and increased proliferation of melanoma cells, this makes PHF5A to an interesting therapeutic candidate.

Although the deficient splicing pattern does not occur on a global level, we were able to observe strong effects on the melanoma cells like reduced clonogenicity and viability of melanoma cells after the knockdown of PHF5A. One of the most characteristic features of malignant melanoma cells is the ability to form colonies originating from one single cell. Together with abnormal cell proliferation, typical for cancer cells, these are two very important properties we were able to inhibit by a knockdown of PHF5A. Fundamentally, each investigated melanoma cell line, especially WM1158, showed an increased apoptosis rate after the RNAi‐mediated knockdown of PHF5A. Further investigating this observation, we were able to show that the loss of PHF5A results in dysregulation of splicing of the anti‐apoptotic gene *FASTK*, which has already been shown in breast cancer cells.^19^ Since the abnormal splicing event contains a retention of intron 5, this provokes a premature stop codon and therefore leads to an aberrant version of the *FASTK* transcript, strongly suggesting that this leads to the abortion of translation or at least a loss‐of‐function‐protein. Confirming this hypothesis, a study conducted by Li et al.^57^ showed that an RNAi‐mediated knockdown of endogenous FASTK is followed by apoptosis, whereas overexpression inhibits this effect. Hubert et al. could also link the loss of PHF5A to inhibited splicing of numerous genes, leading to cell cycle arrest and loss of viability.^20^

Further investigating the apoptotic phenotype after the loss of PHF5A in melanoma cells, our study revealed that the knockdown of PHF5A leads to an activation of the unfolded protein response (UPR) via PERK and ATF6‐signalling pathways with an increased expression of C/EBP Homologous Protein (CHOP). To further underline the observed UPR activation, we were able to detect increased accumulation of protein aggregations after siPHF5A. UPR is the response of cells to the endoplasmic reticulum (ER) stress as a consequence of the enrichment of unfolded proteins in the lumen and is thought to be a characteristic of tumour cells.^58^ To fulfil the huge frequency of proliferation, the cells underlie an abnormally increased protein production. This inevitably results in the accumulation of a heightened amount of misfolded or unfolded proteins. For this reason, tumour cells developed UPR as a survival mechanism, which is triggered by this.^59^, ^60^ If the overload of unfolded or misfolded proteins in the ER is not resolved, the prolonged UPR will induce ER stress‐associated apoptosis by inducing the expression of pro‐apoptotic transcription factors ATF4 and CHOP.^40^, ^61^, ^62^, ^63^ In this study, we were able to confirm an upregulation of the key players in UPR: ATF6‐N, phospho‐eIF2α, as well as the major regulators of subsequent apoptosis CHOP and ATF4. We were not able to find the UPR‐specific splicing pattern of the gene *XBP1*, probably because of the defects in splicing caused by the loss of PHF5A. The spliced variant of *XBP1* would encode a transcription factor, involved in the production of ER chaperones.^64^ Based on these findings, we believe that the splicing defects after siPHF5A lead to misfolding of the translated proteins and therefore activation of UPR with a subsequent CHOP expression, leading to an increased apoptosis rate in melanoma cells. This assumption is supported by a Thioflavin T staining, which visualizes an accumulation of misfolded proteins after the knockdown of PHF5A. Besides of that, the loss of PHF5A prevents the correct splicing and thereby translation of XBP1, an important regulator for chaperons, which support the cells in correct protein folding.

With this study, we were able to highlight the importance of PHF5A as part of the splicing machinery for malignant melanoma cells to promote disease progression.

In summary, understanding the role of PHF5A in the spliceosome gives new insights into post‐transcriptional regulation of melanoma cells. Therapeutic strategies targeting PHF5A could aim to induce apoptosis of melanoma cells while wound healing‐promoting fibroblasts are not harmed by this strategy. Further research is needed to uncover the precise splicing mechanisms of PHF5A and downstream signalling pathways involved in this new regulatory network and its therapeutic implications for melanoma treatment.

## AUTHOR CONTRIBUTIONS

T.M., L.L.‐P. and A.K.B.: Conceptualization; T.M., S.S., A.J.A., C.M., J.G. and M.K.‐F.: investigation, methodology; T.M., M.K.‐F., J.G., A.J.A., C.M., A.K.B., L.L.‐P.: formal analyses, validation, visualization; A.K.B.: funding acquisition, supervision, project administration; T.M., A.K.B., S.S., J.G., A.J.A. and C.M.: Writing – Original Draft; T.M., S.S., A.J.A., C.M., J.G., M.K.‐F., L.L.‐P. and A.K.B.: Writing – Review & Editing. All authors have read and agreed to the published version of the manuscript.

## CONFLICT OF INTEREST STATEMENT

The authors declare no competing interests.

## Supporting information


Figures S1–S2.

Data S1.

Data S2.

Data S3.

## Data Availability

The data that support the findings of this study are openly available in Bioproject at https://www.ncbi.nlm.nih.gov/bioproject/, reference number ID PRJNA1044935. The data for Figure 1D are available at the GEPIA database at http://gepia.cancer-pku.cn/index.html. The data for Figure 1E are available at the COSMIC database at https://cancer.sanger.ac.uk/cosmic. For Figure 1G, we used publicly available data from the Protein Atlas Database at https://www.proteinatlas.org/ENSG00000100410-PHF5A/pathology/melanoma. IGV was used for the representation of Figure 4: 10.1093/BIB/BBS017.
