# Supplementary material for: Splicing control by PHF5A is crucial for melanoma cell survival
Source: Cell Prolif. 2024 Aug 30;58(2):e13741. doi: 10.1111/cpr.13741 (PMC11839196; doi:10.1111/cpr.13741)
Supplement: Supplementary file 1 — Figures S1–S2. [file CPR-58-e13741-s002.pdf]

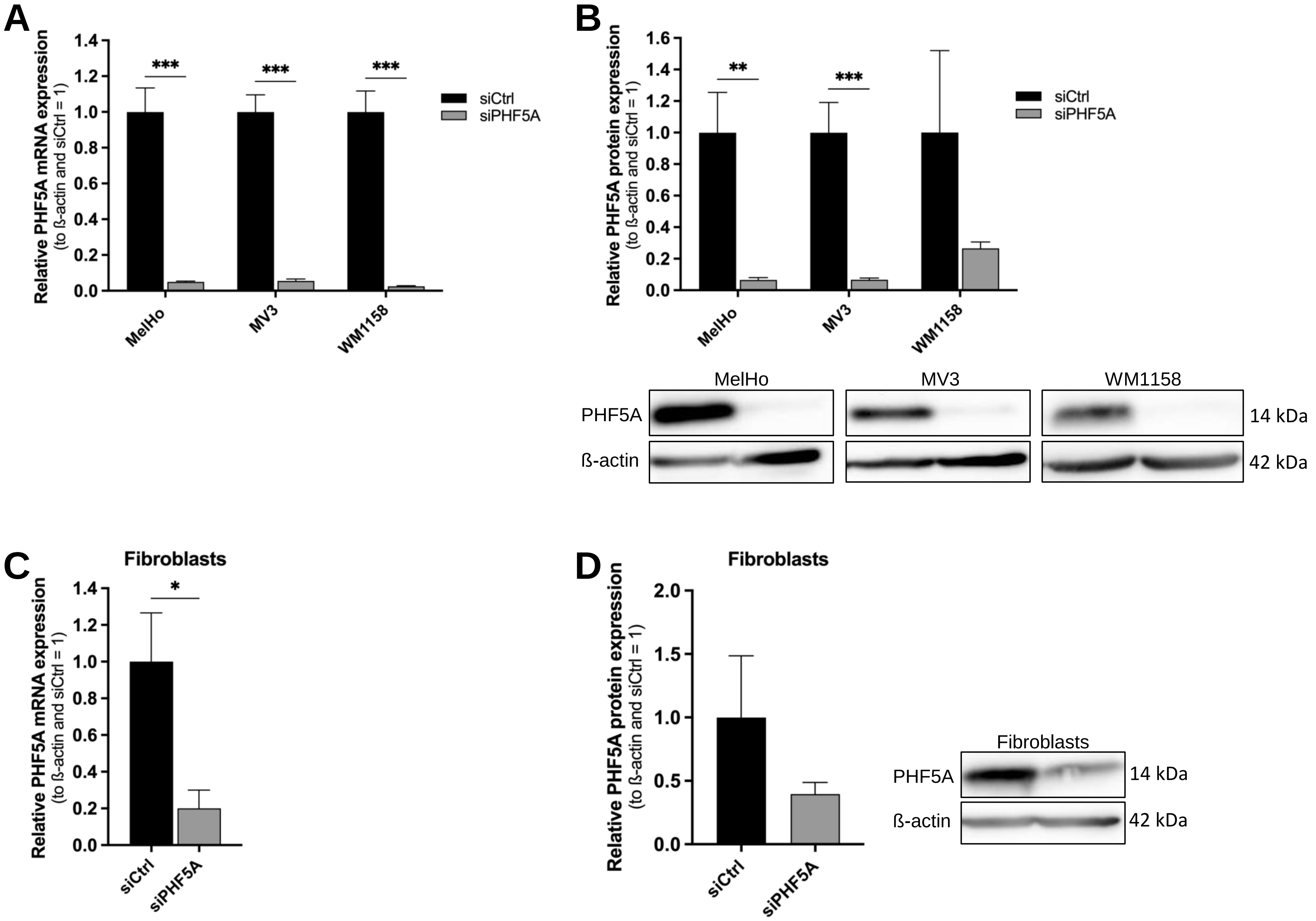

Figure S1. PHF5A knockdown

(**A**) Relative PHF5A mRNA expression to β-actin and siCtrl in MelHo, MV3 and WM1158 (ΔCP), measured with qRT-PCR (n = 10 (WM1158), n = 14 (MelHo), n = 18 (MV3(***p < 0.001, Two-way ANOVA and sub-sequent Bonferroni Multiple Comparison Test). Data are represented in mean ± SEM.

(**B**) Relative PHF5A protein expression to β-actin and siCtrl in MelHo, MV3 and WM1158, analyzed by Western Blot (mean ± SEM, n = 6 (WM1158), n = 10 (MelHo), n = 13 (MV3(*p < 0.05, **p < 0.01, Two-way ANOVA and sub-sequent Bonferroni Multiple Comparison Test). One representative western blot per cell line is shown. Data are represented in mean ± SEM.

(**C**) Relative PHF5A mRNA expression to β-actin and siCtrl in fibroblasts (ΔCP), measured with qRT-PCR (n = 3, one-sample t-test). Data are represented in mean ± SEM.

(**D**) Relative PHF5A protein expression to β-actin and siCtrl in fibroblasts, analyzed by Western Blot (n = 4, one-sample t-test). One representative blot per cell line is shown. Data are represented in mean ± SEM.

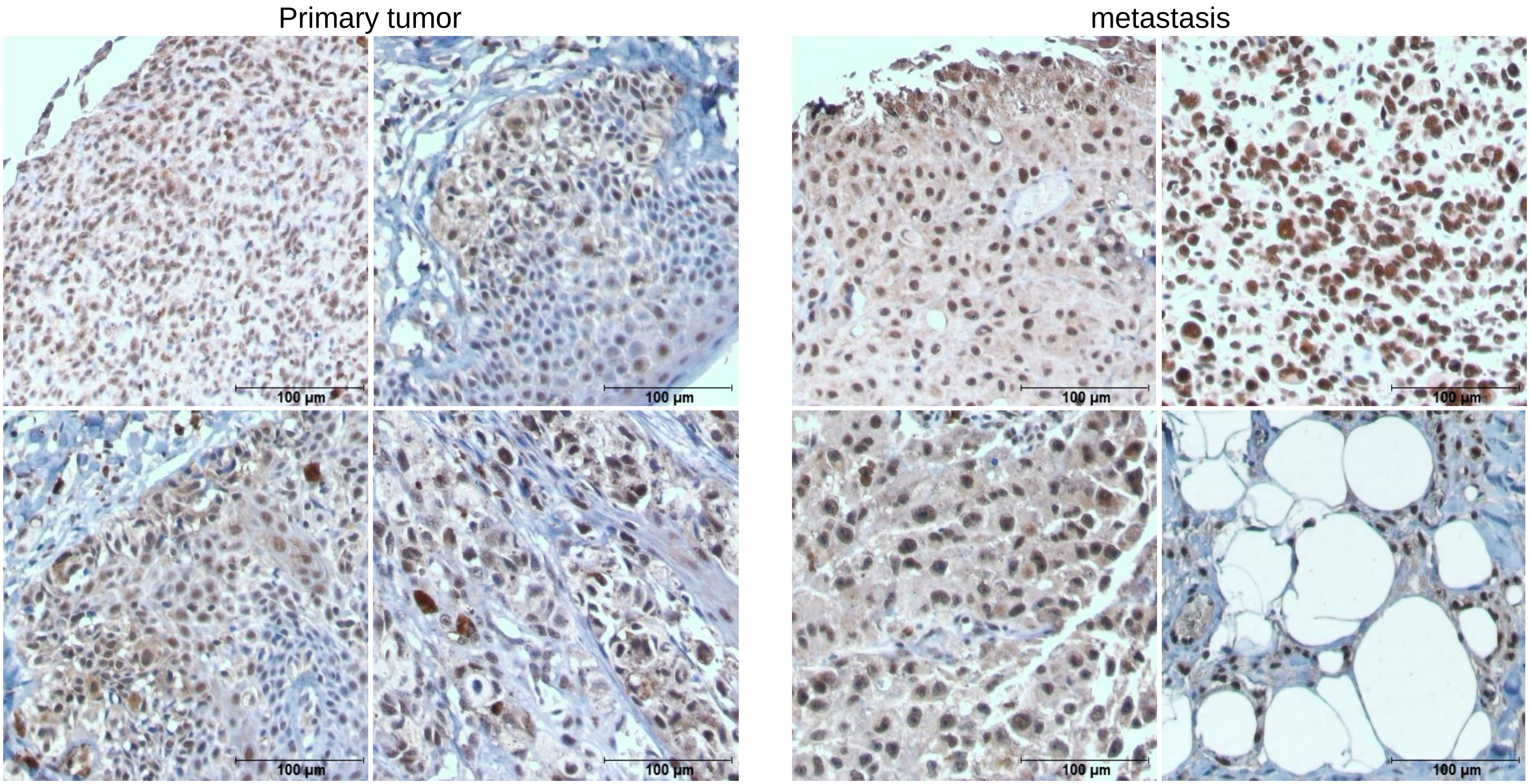

Figure S2. Immunohistochemical staining of PHF5A protein

Exemplary images of additional four human patient derived tumor samples from malignant melanoma primary (left panels) tumor and metastasis (right panels), respectively. Bar shows 100 µm. Stained with antibody against PHF5A in dilution of 1:50.
